# Wnt Inhibition Facilitates RNA-Mediated Reprogramming of Human Somatic Cells to Naive Pluripotency

**DOI:** 10.1016/j.stemcr.2019.10.009

**Published:** 2019-11-07

**Authors:** Nicholas Bredenkamp, Jian Yang, James Clarke, Giuliano Giuseppe Stirparo, Ferdinand von Meyenn, Sabine Dietmann, Duncan Baker, Rosalind Drummond, Yongming Ren, Dongwei Li, Chuman Wu, Maria Rostovskaya, Sarah Eminli-Meissner, Austin Smith, Ge Guo

**Affiliations:** 1 Wellcome–MRC Cambridge Stem Cell Institute, University of Cambridge, Cambridge CB2 0AW, UK; 2 Department of Biochemistry, University of Cambridge, Cambridge CB2 1QW, UK; 3 Guangzhou Institutes of Biomedicine and Health (GIBH), Chinese Academy of Sciences, Guangzhou 510530, China; 4 Department of Medical & Molecular Genetics, King's College London, London SE1 9RT, UK; 5 Institute of Food, Nutrition and Health, ETH Zurich, 8603 Schwerzenbach, Switzerland; 6 Sheffield Diagnostic Genetic Service, Sheffield Children's NHS Foundation Trust, Sheffield S10 2TH, UK; 7 REPROCELL USA, 9000 Virginia Manor Road #207, Beltsville, MD 20705, USA

**Keywords:** RNA-mediated reprogramming, human pluripotent stem cells, Wnt signaling, naive pluripotency, molecular reprogramming, human pre-implantation epiblast, induced pluripotent stem cells

## Abstract

In contrast to conventional human pluripotent stem cells (hPSCs) that are related to post-implantation embryo stages, naive hPSCs exhibit features of pre-implantation epiblast. Naive hPSCs are established by resetting conventional hPSCs, or are derived from dissociated embryo inner cell masses. Here we investigate conditions for transgene-free reprogramming of human somatic cells to naive pluripotency. We find that Wnt inhibition promotes RNA-mediated induction of naive pluripotency. We demonstrate application to independent human fibroblast cultures and endothelial progenitor cells. We show that induced naive hPSCs can be clonally expanded with a diploid karyotype and undergo somatic lineage differentiation following formative transition. Induced naive hPSC lines exhibit distinctive surface marker, transcriptome, and methylome properties of naive epiblast identity. This system for efficient, facile, and reliable induction of transgene-free naive hPSCs offers a robust platform, both for delineation of human reprogramming trajectories and for evaluating the attributes of isogenic naive versus conventional hPSCs.

## Highlights

• Generation of transgene-free human naive iPSCs by RNA-mediated reprogramming
• Wnt inhibition facilitates naive iPSC production and expansion
• Naive iPSCs retain a diploid karyotype
• Naive iPSCs are transcriptomically related to pre-implantation human epiblast

## Teaser

Molecular reprogramming can induce different states of pluripotency. In this paper, Ge Guo and colleagues report that naive stem cells related to the blastocyst stage human embryo can be generated reliably from human somatic cells using RNA delivery of reprogramming factors. Reprogramming is enhanced by inhibition of the Wnt pathway, which also stabilizes the human naive state.

## Introduction

Human pluripotent stem cells (hPSCs) provide a potent resource for fundamental research into early human development and in addition hold great promise for biomedical applications. hPSCs have been derived by culture of explanted human embryo inner cell masses (ICMs) (O'Leary et al., 2012, Thomson et al., 1998) and by reprogramming of somatic cells (Takahashi et al., 2007, Yu et al., 2007). The precise relationship between conventional hPSCs and *in vivo* epiblast development is uncertain, but they have diverged from ICMs (Yan et al., 2013) and appear to represent a post-implantation stage approaching gastrulation (Nakamura et al., 2016). Consequently these cells are often described as primed (Nichols and Smith, 2009, Rossant and Tam, 2017). A second type of hPSC has been isolated more recently using alternative culture conditions based on inhibition of the ERK pathway (Takashima et al., 2014, Theunissen et al., 2014). These cells are termed naive because they show similarities to the pre-implantation epiblast (Guo et al., 2016, Stirparo et al., 2018, Theunissen et al., 2016) and may be analogous to the archetypal embryonic stem cells established in mouse (Nichols and Smith, 2012, Smith, 2001). Naive hPSCs are obtained by resetting the status of conventional hPSCs using transgenes (Takashima et al., 2014) or by culture manipulation (Guo et al., 2017, Theunissen et al., 2014). Naive cell lines can also be established directly from dissociated embryo ICMs (Guo et al., 2016).

Somatic cell reprogramming directed by ectopic transcription factors can generate induced pluripotency (Takahashi and Yamanaka, 2006). The canonical Yamanaka reprogramming factors yield induced pluripotent stem cells (iPSCs) that in mouse are naive, but in human are primed (Okita et al., 2007, Silva et al., 2008, Takahashi et al., 2007). This difference may be determined by the appropriateness of the culture environment for capture of naive versus primed states, respectively. Indeed, mouse primed iPSCs can be obtained by reprogramming in medium containing fibroblast growth factor (FGF) and activin (Han et al., 2011), similar to culture conditions for propagation of conventional hPSCs (Vallier et al., 2005). Induction of naive pluripotency is relatively robust in the mouse system and is increasingly well characterized at the molecular level (Guo et al., 2019, Schiebinger et al., 2019, Stadhouders et al., 2018). Reprogramming of human fibroblasts to naive iPSCs has only recently been reported, however, and appears variable and inefficient (Kilens et al., 2018, Liu et al., 2017). The methods entailed protracted reprogramming factor expression from viral or episomal vectors and the iPSCs frequently exhibited persisting transgenes. Moreover, the reprogrammed cells obtained were heterogeneous with poorly characterized differentiation behavior. Very recently, reprogramming to the human naive state was achieved using chemically modified mRNA vectors applied in a microfluidic apparatus (Giulitti et al., 2019). In that study the authors report that serial transfection with modified mRNAs over at least 7 days within microfluidic chambers are important for induction of naive cells. Such findings for human naive reprogramming contrast with observations in the mouse in which naive iPSCs are readily obtained by multiple methods requiring only short-term exposure to reprogramming factors in standard tissue culture conditions.

Here we sought to determine whether human naive iPSCs could be produced directly from somatic cells in bulk culture with simplicity and efficiency comparable to the generation of mouse iPSCs. Integration and/or persisting expression of reprogramming factor transgenes is undesirable in principle, and specifically may perturb the naive PSC state or subsequent differentiation. We therefore focused on producing transgene-free naive hPSCs by transient delivery of non-modified RNAs (Poleganov et al., 2015).

## Results

### RNA-Mediated Induction of Naive Pluripotency Is Facilitated by Inhibition of the Canonical Wnt Pathway

RNA-directed reprogramming has previously been used to generate conventional human iPSCs (Poleganov et al., 2015). We reasoned that the same system may induce naive pluripotency under the appropriate culture conditions. We adopted the combination of mRNAs encoding six reprogramming factors, OCT4, SOX2, KLF4, c-MYC, NANOG, and LIN28 (OSKMNL), augmented with microRNAs 302 and 367, plus Vaccinia virus immune evasion factors E3, K3, and B18R mRNAs to suppress the interferon response. Naive hPSCs were originally established and propagated in medium containing the MEK1/2 inhibitor PD0325901, the glycogen synthase kinase-3 (GSK3) inhibitor CH99021, the atypical protein kinase C inhibitor Gö6983, and the cytokine leukemia inhibitory factor (LIF), collectively termed t2iLGö (Guo et al., 2016, Takashima et al., 2014). More recently, however, we have found that the tankyrase inhibitor and Wnt pathway antagonist XAV939 (XAV) enhances transgene-free resetting of conventional PSC to naive status (Bredenkamp et al., 2019, Guo et al., 2017). Therefore, we examined the respective effects of CH and XAV during RNA-mediated reprogramming.

**Figure 1:**
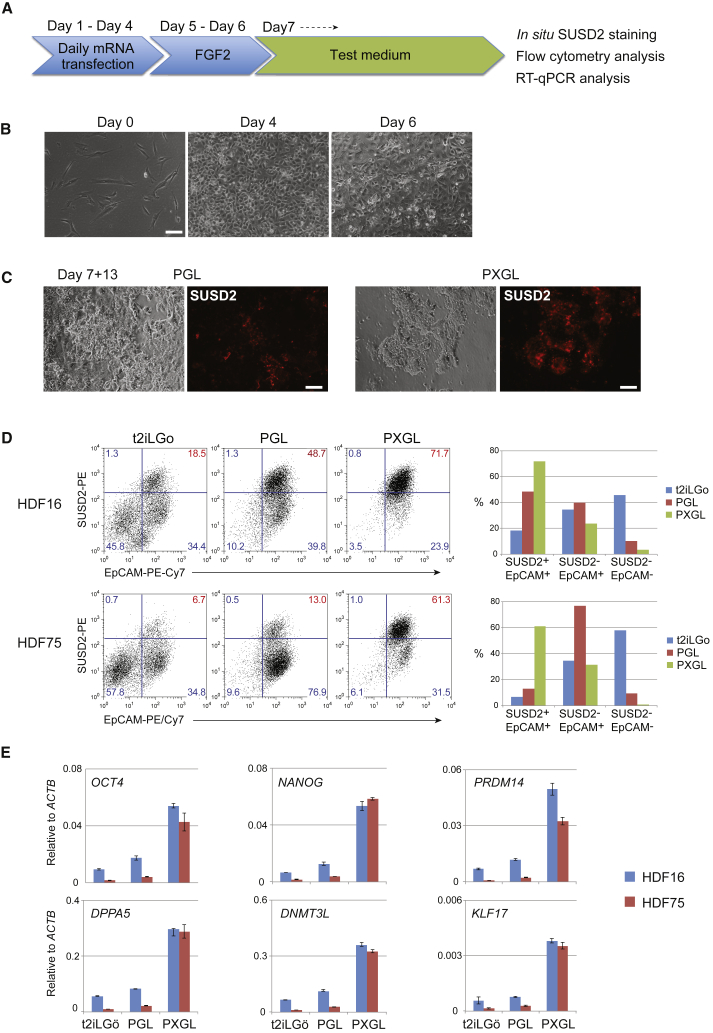
Wnt Inhibition Enhances Naive Reprogramming by RNA (A) Schematic of reprogramming protocol. (B) Morphology during initial reprogramming in medium with FGF2. (C) Morphology in naive capture medium, PGL or PXGL. See also Figure S1A. (D) Flow cytometry analysis of EpCAM and SUSD2 expression after 12 days in PGL with CH (t2iLGö) or XAV. Scatterplots on left, histograms on right. (E) qRT-PCR analysis of pluripotency markers after 12 days in PGL-based medium. Scale bars, 100 μm. Error bars indicate SD of two technical replicates. See also Figure S1.

We plated 10,000 human dermal fibroblasts (HDFs) on Geltrex-coated four-well tissue culture plates and after overnight incubation carried out transfections with the RNA cocktail for 4 consecutive days (Figure 1A). Cells were then cultured in medium containing FGF2 for 2 days before exchange to naive reprogramming media. The naive media each contained PD0325901 (1 μM), Gö6938 (2 μM), and human LIF (10 ng/mL), plus the Rho-associated kinase inhibitor Y27632 (1 μM). To this base medium, termed PGL, we added either CH (1 μM), as in the original t2iLGö naive hPSC culture formulation (Takashima et al., 2014), or XAV (2 μM), constituting PXGL. Fibroblasts grew to a near-confluent layer of cells after 4 days of mRNA cocktail transfection. Patches of cells undergoing mesenchymal to epithelial transition became apparent from day 6 (Figures 1B and S1A). Following transfer to PGL-based naive media we observed compact colonies of cells with smooth boundaries after a further 10 days (Figure S1A). Presence of XAV resulted in markedly more of these colonies and a corresponding reduction in alternative cell morphologies.

Sushi domain containing 2 (SUSD2) is a cell surface protein highly expressed by human pre-implantation epiblast cells and naive hPSCs (Bredenkamp et al., 2019). By *in situ* live staining we detected expression of SUSD2 on the majority of compact colonies in reprogramming cultures in the presence of XAV (Figures 1C and S1A). We quantified the effect of XAV or CH by flow cytometry using SUSD2 together with the pan-epithelial marker EpCAM. The proportion of SUSD2+EpCAM+ cells was substantially higher in the presence of XAV than in PGL. Conversely, CH reduced the number of SUSD2+EpCAM+ cells (Figure 1D). Consistent with SUSD2 analysis, cultures reprogrammed in the presence of XAV showed substantially higher expression of core pluripotency factors and of naive markers assayed by qRT-PCR (Figure 1E).

Tankyrase inhibition blocks canonical Wnt signaling but may also affect other pathways. We therefore evaluated RNA reprogramming in PGL supplemented with IWP2, a PORCN inhibitor, which blocks Wnt signaling by inhibition of Wnt protein secretion (Chen et al., 2009). Similar to XAV, addition of IWP2 yielded an increased proportion of SUSD2+EpCAM+ cells (Figures S1B and S1C). The culture expressed higher levels of naive markers than cells reprogrammed in non-supplemented PGL (Figure S1D). We noted reduced expression of Wnt target genes *AXIN1* and *TBX3* in presence of XAV or IWP2 (Figure S1D).

### Reproducibility of Reprogramming to Naive Status

**Figure 2:**
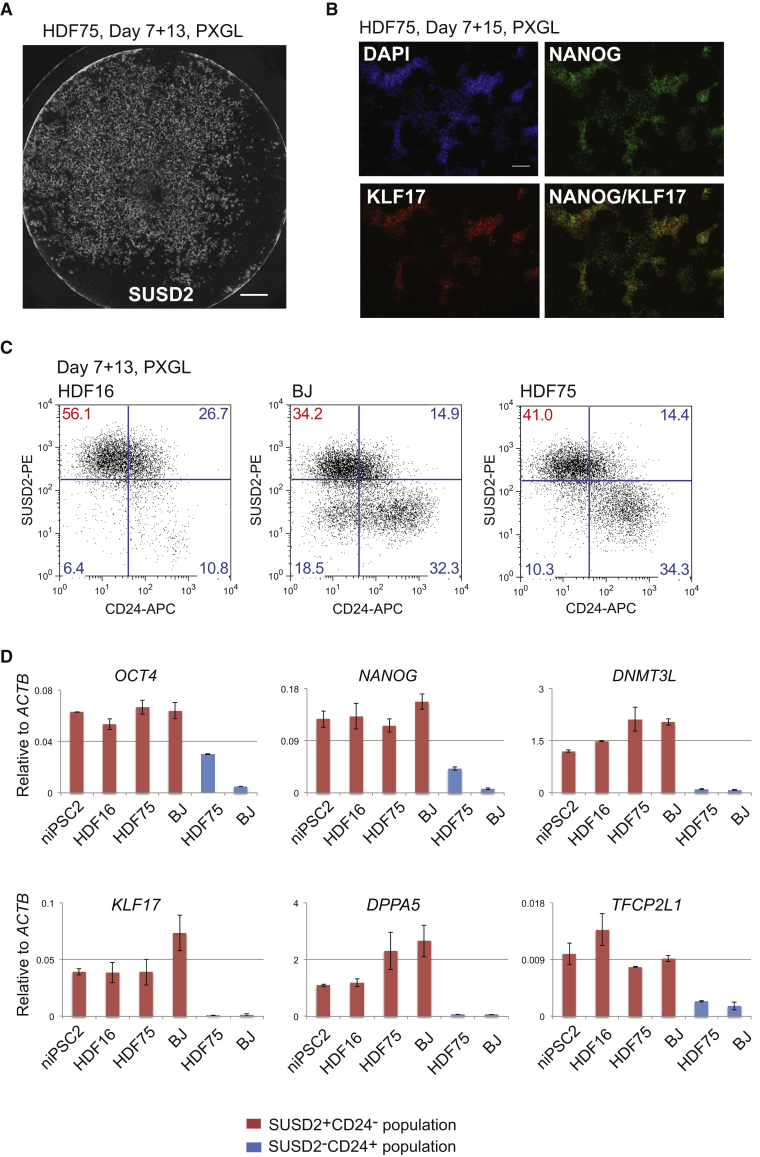
Reproducibility of Reprogramming in PXGL (A) Well of HDF75 reprogramming culture after 13 days in PXGL, stained *in situ* with SUSD2-PE antibody. See also Figure S1B. Scale bar, 2 mm. (B) Immunostaining for KLF17 and NANOG after 15 days in PXGL. Scale bar, 100 μm. (C) Flow cytometry analysis SUSD2 and CD24 expression at day 13 in PXGL for different fibroblast lines. (D) Marker analysis by qRT-PCR of isolated SUSD2+ and SUSD2– populations. Error bars indicate SD of two technical replicates. See also Figure S2.

Somatic cell reprogramming can vary between cell lines. To evaluate reproducibility of RNA-directed reprogramming to a naive phenotype we applied the protocol using PXGL to two adult primary dermal fibroblasts (HDF16 and HDF75) and one newborn foreskin fibroblast (BJ). The experiments were repeated at different passages and we tested three different batches of RNA cocktail. In all cases we obtained SUSD2+ colonies. SUSD2 live staining after 12–14 days in PXGL typically revealed several hundred stained colonies per well of a 4-well plate (Figures 2A and S2A). To substantiate the character of these colonies we performed immunostaining for diagnostic transcription factors. KLF17 is a transcription factor expressed in the early human embryo and in naive PSCs but completely absent from conventional PSCs (Blakeley et al., 2015, Guo et al., 2016), and NANOG is a critical pluripotency factor expressed in both naive and conventional hPSCs. We detected co-expression of KLF17 and NANOG proteins in the majority of reprogrammed colonies in PXGL (Figures 2B and S2B).

Human naive and conventional PSCs are distinguished by differential expression of SUSD2 or CD24 surface markers, respectively (Bredenkamp et al., 2019). Accordingly, we quantified naive reprogramming for HDF16, HDF75, and BJ cultures based on presence of SUSD2 and absence of CD24 after 14 days in PXGL (Figure 2C). For HDF16, more than half of the culture (56%) was composed of SUSD2+CD24– cells. BJ and HDF75 cells were more mixed at this stage; In addition to SUSD2+ cells, a distinct SUSD2–/CD24+ population was also present. We purified these two populations and subjected them to qRT-PCR analysis. SUSD2+ cells express naive markers KLF17, KLF4, TFCP2L1, DPPA5, and DNMT3L, while the CD24+SUSD2– populations express general pluripotency markers OCT4 and NANOG at low levels but lack naive hallmarks (Figure 2D).

We performed parallel RNA reprogramming of HDF16 and HDF75 to primed or naive iPSC status (Figures S2C and S2D). The primed PSC surface marker CD24 was expressed on >50% of cells 4 days after transfer to E7 medium (Figure S2E). During naive reprogramming in PXGL, SUSD2 expression appeared later, but reached a similar final proportion.

We then investigated reprogramming of an alternative somatic cell type, peripheral blood-outgrowth derived endothelial progenitor cells (EPCs) (Geti et al., 2012). EPC reprogramming requires more prolonged RNA transfection over 8 days (Poleganov et al., 2015), during which there is considerable cell death (Figure S3A). Surviving cells were transferred to PXGL and, after 3 weeks, we observed occasional patches of compact epithelial cells. A low iPSC yield compared with fibroblasts has previously been noted during reprogramming of EPCs to primed iPSCs (Poleganov et al., 2015). About 5–6% of surviving cells were positive for SUSD2 and EpCAM, and negative for CD24 (Figure S3B), indicative of naive status (Bredenkamp et al., 2019).

### Expansion of Naive iPSCs Generated by RNA-Mediated Reprogramming

**Figure 3:**
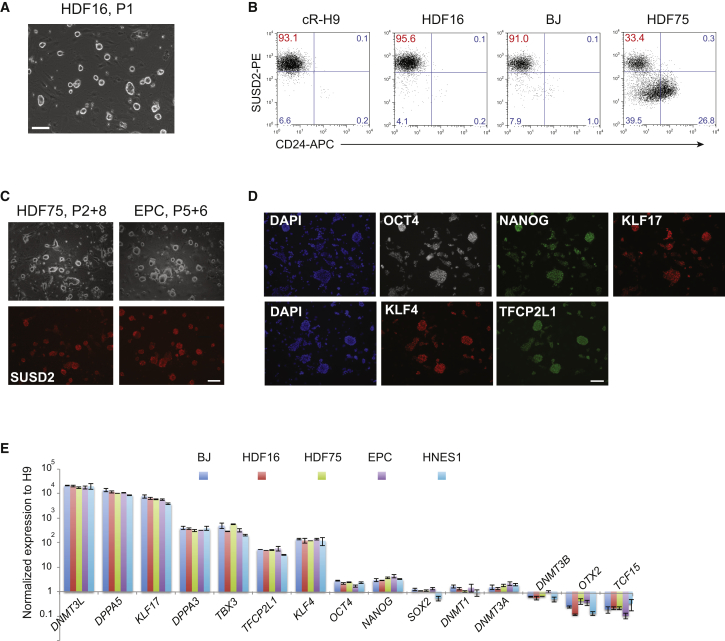
Expansion and Characterization of Naive iPSCs (A) Morphology of naive iPSC culture on MEFs at passage 1 after reprogramming. (B) Flow cytometry analysis of SUSD2 and CD24 expression in HDF16-, HDF75-, and BJ-derived naive iPSC cultures at passage 2, compared to chemically reset H9 naive cells. (C) SUSD staining of naive iPSC cultures of indicated origin after sorting and subsequent passaging (P). (D) Immunostaining for naive markers in expanded naive iPSCs (BJ derived). (E) qRT-PCR analysis of marker expression in expanded naive iPSCs of indicated origins and embryo-derived naive HNES1 cells. Data are normalized to expression in conventional H9 cells. Error bars indicate SD of two technical replicates. Scale bars, 100 μm. See also Figure S3.

After 14 days in PXGL for HDFs and 21 days for EPCs, we bulk passaged cultures via dissociation with Accutase and replated onto feeder layers of mouse embryo fibroblasts (MEFs) in PXGL plus ROCK inhibitor. Dome-shaped, refractile, colonies formed on MEFs (Figure 3A). After two passages we obtained cultures with more than 90% SUSD2+ cells from HDF16 and BJ (Figure 3B). HDF75- and EPC-derived cultures remained heterogeneous. In these cases we used flow cytometry to purify the SUSD2+/CD24– population. Thereafter we found that cells could readily be maintained with relatively homogeneous naive colony morphology and SUSD2 expression (Figure 3C). Cultures were passaged every 4–5 days at a 1:3 or 1:5 split ratio for at least 6 weeks (>10 passages). Expanded cultures display naive transcription factor proteins KLF17, NANOG, KLF4, and TFCP2L1 (Figures 3D and S3). qRT-PCR analysis showed expression of naive markers at comparable levels to naive HNES cells derived from dissociated human ICMs (Guo et al., 2016) (Figure 3E). We generated naive iPSCs from two further EPC lines and established stable lines by both SUSD2 sorting and bulk passaging. These cells expressed naive markers at comparable levels with HDF-derived naive iPSCs (Figure S3D).

**Figure 4:**
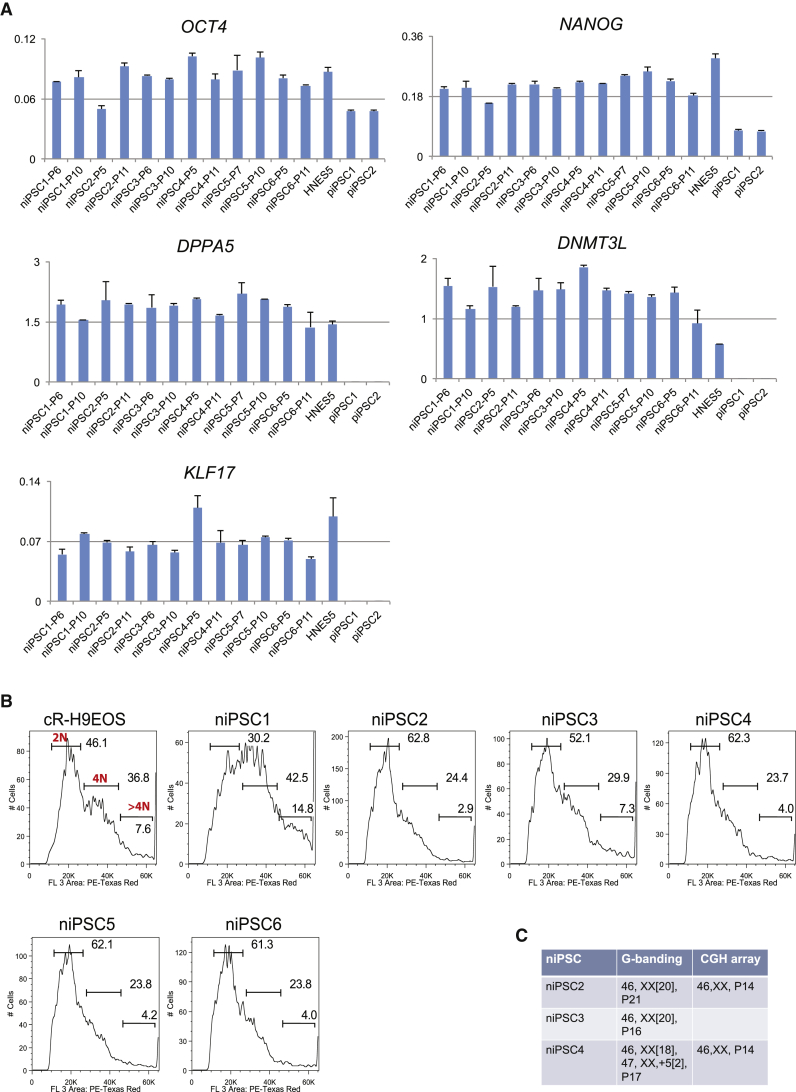
Expansion of Naive iPSCs from Single Colonies (A) qRT-PCR analysis of pluripotency markers in six expanded naive iPSC colonies at indicated passages. Two isogenic conventional iPSC colonies (piPSC1 and piPSC2) expanded in parallel and embryo-derived HNES5 cells are included for comparison. Error bars indicate SD of two technical replicates. (B) DNA content analysis from flow cytometry profiles of cells stained with propidium iodide. Diploid genome population is labeled as 2N, 4N indicates cells in G2 and/or tetraploid, hyperpolypoid is >4N. (C) Chromosome analyses of expanded niPSC colonies at indicated passasages (P).

We also investigated expansion of individual colonies from the primary reprogramming well. We manually picked eight colonies from HDF75 cultures after 14 days in PXGL. Colonies were dissociated with Accutase and plated in PXGL plus ROCK inhibitor on MEFs in a 96-well plate. Six colonies were expanded into stable naive iPSC cultures that maintained naive marker gene expression (Figure 4A).

We previously noted incidences of polyploidy in naive cells cultured in t2iLGö medium (Guo et al., 2016). Therefore, we monitored DNA content in the expanded naive iPSC colonies by propidium iodide staining and flow cytometry analysis. One line, niPSC1, contained a fraction of hyperdiploid cells at passage 5 but the other five remained diploid at passage 10 (Figure 4B). We performed G-banding karyotype analysis on three diploid lines, niPSC2, niPSC3, and niPSC4, after further expansion. Two lines, niPSC2 and niPSC3, exhibited a normal 46XX diploid karyotype at passages 20 and 16, respectively (Figure 4C). The third line, niPSC4, was predominantly diploid but with a subpopulation (10%) of cells showing trisomy for chromosome 5 at passage 17. Array comparative genomic hybridisation (CGH) analysis did not detect any large copy number variations (CNVs) in two clones examined (Figure 4C). Collectively these data indicate that human naive iPSCs can be generated and expanded in PXGL with a relatively stable chromosome content.

### Somatic Lineage Differentiation of Naive iPSCs

**Figure 5:**
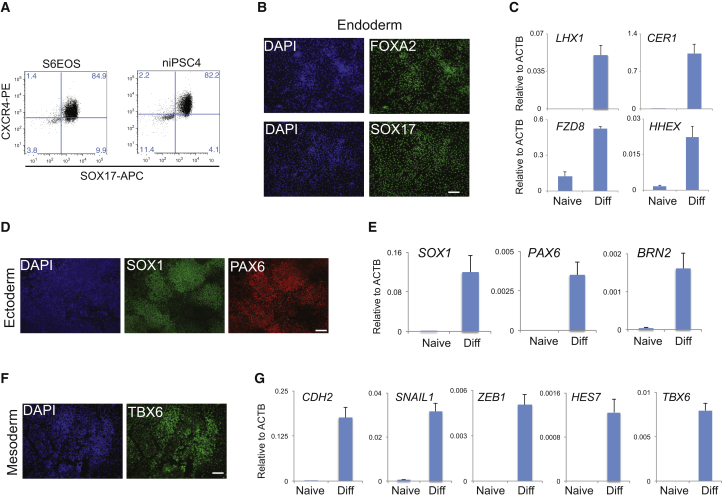
Differentiation of Capacitated Naive iPSCs (A) Flow cytometry analysis of SOX17 and CXCR4 expression after 3 days definitive endoderm induction of primed S6EOS and capacitated niPSC4 cells. (B) Immunostaining for FOXA2 and SOX17 after 3 days definitive endoderm induction of niPSC2. (C) qRT-PCR analysis of definitive endoderm markers after 3 days induction of niPSC2. (D) Immunostaining for SOX1 and PAX6 after 10 days neuroectoderm induction of niPSC2. (E) qRT-PCR analysis of neuroectoderm marker expression. (F) Immunostaining for TBX6 after 6 days of paraxial mesoderm induction of niPSC2. (G) qRT-PCR analysis of paraxial mesoderm markers. Scale bars, 100 μm. Error bars indicate SD of three technical replicates. See also Figure S4.

Naive PSCs are related to pre-implantation epiblast and consequently are not directly competent for somatic lineage induction (Rostovskaya et al., 2019, Smith, 2017). Formative transition of human naive PSCs can be achieved by transfer to N2B27 medium supplemented with XAV, a process termed capacitation (Rostovskaya et al., 2019). We examined differentiation potential of niPSC2 and niPSC4 following 13 days capacitation. Capacitated HNES1 cells and isogenic primed iPSCs were included for comparison. Both naive iPSC clones differentiated efficiently to definitive endoderm, neuroectoderm, and paraxial mesoderm on directed lineage induction. For definitive endoderm, we quantified co-expression of SOX17 and CXCR4 in more than 80% of cells after 3 days by flow cytometry (Figure 5A). For each of the induced lineages, marker expression was detected by qRT-PCR and immunostaining (Figures 5B–5G) at comparable levels as for capacitated HNES1 and primed iPSC differentiation (Figures S4A–S4C). We also assessed directed differentiation after capacitation from niPSC populations generated from HDF75, HDF16, and EPCs. In each case appropriate lineage markers were induced (Figures S4D–S4E).

### Global Transcriptome and DNA Methylome Features of Naive Human iPSCs

**Figure 6:**
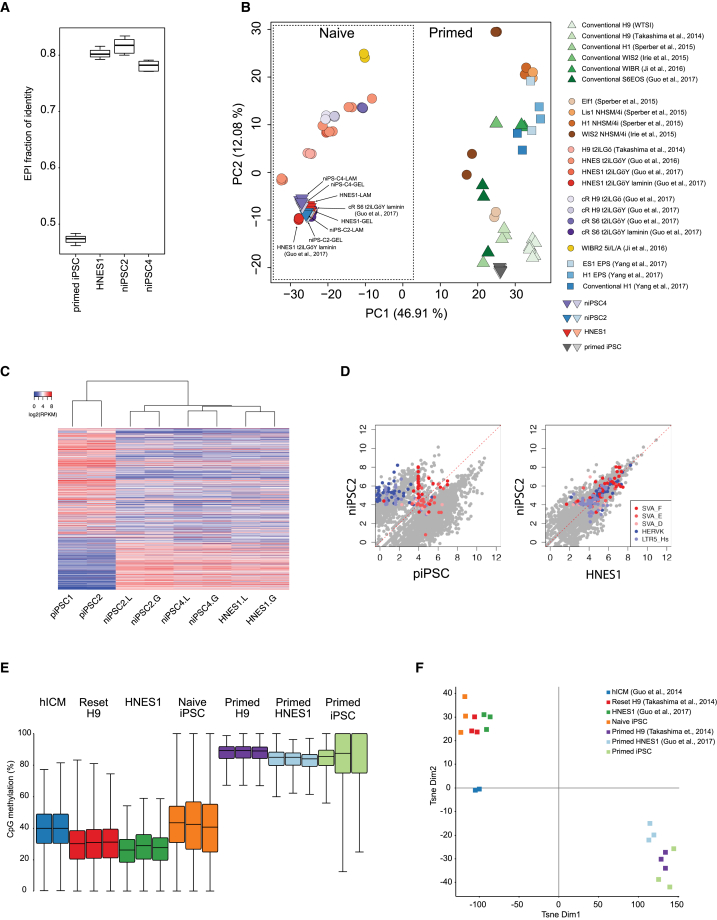
Global Molecular Analyses of Naive iPSCs (A) Fraction of identity with human pre-implantation epiblast for primed iPSCs, embryo-derived naive stem cells (HNES1), and naive iPSCs. Boxplots show four independent cell cultures of each indicated type. (B) Principal-component analysis using all expressed protein-coding genes. (C) A heatmap showing the expression of 6,290 differentially expressed TEs (log2FC > 2, p < 0.05 in any pairwise comparison; and log2(norm counts) > 3.5 expression in any sample). TEs are ranked by the average log2FC of four possible different comparisons between naive iPSC (niPSC) on laminin (L) or geltrex (G), and primed iPSC (piPSC) cell types. (D) Scatterplots showing the expression of TEs in piPSCs, niPSC2, and HNES1 cells. TEs from representative TE subfamilies that are differentially expressed between naive and primed cells are highlighted. (E) Boxplots showing the global distribution of CpG methylation levels from pooled replicates of the indicated samples compared with published datasets (Guo et al., 2014, Guo et al., 2017, Takashima et al., 2014). iPSC samples are from two independent experiments. Methylation was quantitated over 20-kb genomic tiles. (F) tSNE plot showing the distribution and clustering of the analyzed datasets. Methylation was quantitated over 20-kb genomic tiles. See also Figure 5.

We carried out RNA sequencing (RNA-seq) on niPSC2, niPSC4, and HNES1 cells passaged in PXGL on either Geltrex or laminin to exclude MEFs. Two primed iPSC cultures generated by RNA-mediated reprogramming were examined in parallel. We applied quadratic programming (DeconRNAseq) to assess quantitatively the similarity between the PSC cultures and human pre-implantation development based on global transcriptome profiles (Gong and Szustakowski, 2013, Stirparo et al., 2018). HNES1, niPSC2, and niPSC4 have a median epiblast fraction of identity of 0.8, 0.81, and 0.78, respectively (Figure 6A). These values indicate very high resemblance to pre-implantation epiblasts compared with other stages (zygote, 4-cell, 8-cell, compacted morula, early ICM, and primitive endoderm). In contrast the primed iPSCs show less than 50% fraction of identity to epiblast.

We then compared these samples with other hPSC samples. Dimensionality reduction by principal-component analysis highlights that the naive iPSC clones are very closely related to one another and to HNES1 cells cultured in PXGL, and also to naive PSCs cultured in a previous study in t2iLGö on laminin (Guo et al., 2017) (Figure 6B). Naive PSC cultures on MEFs in t2iLGö (Guo et al., 2016, Guo et al., 2017, Takashima et al., 2014) or 5iLA (Theunissen et al., 2014) are more dispersed but reside in the same major cluster that is unambiguously separated on PC1 from conventional or other hPSC cultures.

A large number of transposable elements (TEs) are differentially expressed between human naive and primed ESCs (Guo et al., 2017, Theunissen et al., 2016). Subgroups of hominid-specific HERVK, LTR5-Hs, and SVA are significantly upregulated in HNES and chemically reset (cR) naive cells, while HERVH and LTR-7 are mostly suppressed. We performed differential expression analysis of TEs between naive and primed iPSCs. Naive iPSCs clustered together with HNES cells and apart from primed iPSCs (Figure 6C). Consistent with our previous observation, HERVK, LTR5-Hs, and SVA-F families are upregulated in naive iPSCs compared with primed iPSCs (Figures 6D, S5A, and S5B).

Naive hPSCs have been found to be globally hypomethylated (Takashima et al., 2014, Theunissen et al., 2016), in common with mouse and human ICM cells (Guo et al., 2014, Lee et al., 2014, Smith et al., 2014). To evaluate genome methylation in naive iPSCs, we performed whole-genome bisulfite sequencing. Methylation profiles for naive and primed iPSCs generated by RNA reprogramming were compared with published datasets for primed hPSCs, human ICM cells (Guo et al., 2014), transgene reset naive PSCs (H9-NK2; Takashima et al., 2014) and HNES1 cells (Guo et al., 2016). The primed iPSCs showed high levels of DNA methylation (85%–95%), as expected. In contrast, naive iPSCs were globally hypomethylated to levels comparable with ICM cells but slightly higher than previously analyzed cultures of transgene reset or embryo-derived hPSCs (Figure 6E). Using t-distributed stochastic neighbor embedding (tSNE) analysis (van der Maaten and Hinton, 2008), we found that methylation profiles of naive and primed PSC cultures clustered apart, with naive cultures adjacent to ICM samples (Figure 5F).

We previously showed that the genome of naive PSCs is not uniformly hypomethylated, and exhibits a small number of regions that gain methylation compared with primed PSCs (Guo et al., 2017). Therefore, we asked whether naive iPSCs displayed similar characteristics. We defined genomic regions (blue) which showed >10% hypermethylation between reset and primed H9-NK2 PSCs (Takashima et al., 2014) and <30% methylation in primed conditions and examined their methylation state in the current datasets (Figure S5C). We found that a substantial number of these regions were also hypermethylated in naive iPSCs, indicating that they may be a specific feature of naive stem cells.

We also assessed the methylation status of imprinted regions in the different iPSC cultures. As observed previously (Guo et al., 2017, Pastor et al., 2016), naive conditions failed to preserve imprinted methylation, although a significant number of imprints also appeared to be eroded in primed iPSCs (Figure S5D).

## Discussion

The findings in this study establish that human somatic cells can be reprogrammed efficiently to the naive PSC state by transient delivery of reprogramming factors using RNA transfection. Thereafter, naive cells can reliably be expanded into stable diploid cell lines, either as bulk populations, by sorting for SUSD2 expression, or by picking individual colonies. Resulting naive iPSC lines exhibit a consistent marker phenotype that is in common with previously characterized naive hPSCs produced by resetting or derived from embryos. Following formative transition, naive iPSCs display competence for differentiation into somatic lineages. Both transcriptome and DNA methylome of naive iPSCs show high global correlation with embryo-derived naive HNES cells and a corresponding relatedness to epiblast cells in the human blastocyst.

Recent studies reported that human naive iPSCs can be generated by transgene-induced reprogramming but that the products may be heterogeneous and confounded by persisting transgenes (Kilens et al., 2018, Liu et al., 2017). Transgene-free naive iPSCs have also been produced using chemically modified RNAs, but the efficiency of this approach was reported to depend on cell confinement in a microfluidic chamber (Giulitti et al., 2019), which restricts general application. In contrast, our results demonstrate that reprogramming to the naive state can be highly efficient using unmodified RNAs in standard cell culture conditions. For dermal fibroblasts, three or four daily transfections with mRNAs encoding OSKMNL reprogramming factors together with miRNAs 302 and 367 are sufficient to produce more than 100 SUSD2+ naive iPSC colonies starting from 10,000 cells in a single well of a 4-well plate. This result is qualitatively reproducible between three different human fibroblast cultures, although individual efficiency varies, as has been generally reported for human reprogramming. Of note, PXGL medium not only promotes establishment of naive pluripotency, but is also relatively selective against other cell types. Consequently most non- or incompletely reprogrammed cells die or growth arrest in these conditions, allowing naive iPSC cultures to be established by bulk passaging without need for colony picking or cell sorting, although both can also be deployed. Occasionally we noticed high levels of cell death during RNA transfection, in which case limiting the transfection period to 3 days preserves viability, and naive colonies are still generated in recoverable numbers. In the case of EPCs, sustained transfection is required and reprogramming efficiency is lower, as also noted for conventional iPSC generation (Poleganov et al., 2015), but sorting for SUSD2+CD24– cells effectively purifies the naive cell fraction and enables subsequent stable expansion.

We found that supplementation with XAV markedly improves the efficiency of reprogramming to the naive state, in line with observations during resetting of conventional PSC (Guo et al., 2017). This may be a key difference from previous reports that found low efficiency of naive reprogramming using media that typically included the GSK3 inhibitor CH (Giulitti et al., 2019, Kilens et al., 2018, Liu et al., 2017). Our analysis shows that the presence of CH inhibits reprogramming to naive status. CH has the opposite effect to XAV or IWP2 of stimulating rather than suppressing canonical WNT signaling. We surmise that blockade of WNT signaling reduces activation of gene expression that can derail reprogramming and/or destabilize naive hPSCs, as demonstrated during resetting (Guo et al., 2017). Thus insulation from WNT signaling appears beneficial for stabilization of naive pluripotency during induction and expansion. This is in line with the general proposition that naive PSC are sustained primarily by preventing differentiation (Martello and Smith, 2014), although differs in detail from the mouse ground state system (Ying et al., 2008). The species difference may largely be explained by the fact that human naive PSC, and *in vivo* human naive epiblast cells, show very low expression of TCF3 (*TCF7L1*) and do not express ESRRB (Rostovskaya et al., 2019, Takashima et al., 2014), the key components regulated by GSK3 inhibition in mouse ESCs (Martello et al., 2012, Wray et al., 2011). In general, we find that stem cell cultures in PXGL exhibit equivalent naive features to cells in our original t2iLGö formulation (Takashima et al., 2014), but appear more robust and stable.

Overall, these analyses establish that human naive iPSCs generated by RNA-directed reprogramming are essentially indistinguishable globally from naive PSCs derived from human ICMs or generated by resetting of conventional hPSCs and are similarly closely related to human pre-implantation epiblast. Relatively facile but reliable generation of naive iPSCs will open up the fields of human reprogramming and naive pluripotency for deeper investigation. In mouse it is well established that somatic cell reprogramming converges on the naive PSC phenotype unless specific culture conditions are applied to capture primed pluripotency (Han et al., 2011). In human, however, the same reprogramming factors as used in mouse routinely generate PSCs of the primed phenotype. Our findings substantiate the hypothesis that the final state of pluripotency obtained by molecular reprogramming is determined in humans as in mice by the culture environment. We speculate that reprogramming to the naive state may be direct in the PXGL culture environment and not entail passage through a primed state. This may be examined by delineating the trajectories of RNA-mediated reprogramming to naive or primed endpoints. The combination of high efficiency with limited duration of reprogramming factor expression makes the mRNA delivery system attractive for such studies using primary cells. Furthermore, as illustrated in the case of XAV, it is straightforward to combine small molecules with mRNA reprogramming and screen for accelerated or enhanced reprogramming, which can readily be visualized and quantified using SUSD2 live staining or flow cytometry (Bredenkamp et al., 2019). Finally, the ability to generate naive iPSCs rapidly and reliably from somatic cells provides a platform for comprehensive evaluation of the consistency, genomic stability, differentiation propensity, and other attributes of naive hPSCs compared with isogenic conventional hPSCs generated from the same donor.

## Experimental Procedures

### Human PSC Culture

Naive hPSCs, including cR, embryo-derived (HNES1), and naive iPSCs were propagated in N2B27 with PXGL (1 μM PD0325901 [P], 2 μM XAV939 [X], 2 μM Gö6983 [G], and 10 ng/mL human LIF [L]) on irradiated MEF feeders. ROCK inhibitor (Y-27632) and Geltrex (0.5 μL/cm^2^ surface area; hESC-Qualified, Thermo Fisher Scientific, A1413302) were added to medium during replating. Cells were cultured in 5% O_2_, 7% CO_2_ in a humidified incubator at 37°C and passaged by dissociation with Accutase (BioLegend, 423201) every 3–5 days. For capacitation, cells were passaged once without feeders in PXGL medium then exchanged into N2B27 containing 2 μM XAV (Rostovskaya et al., 2019). Conventional hPSC cultures were propagated on Geltrex in Essential 8 (E8) medium made in-house (Chen et al., 2011) or AFX medium (N2B27 basal medium with 5 ng/mL activin A, 5 ng/mL FGF2, and 2 μM XAV). Cell lines were maintained without antibiotics and confirmed free of mycoplasma contamination by periodic in-house PCR assay.

### Somatic Cell Culture

Adult HDFs (HDFa), HDFa16, HDFa75 (Thermo Fisher Scientific, C0135C), and BJ foreskin fibroblast (ATCC, CRL-2522) were cultured in DMEM high glucose (Merck, D5546) with FBS (10%, Merck, F0804), L-glutamine (2 mM, Thermo Fisher Scientific, 25030024) and 2-mercaptoethanol (100 μM, Merck, M3148) on 0.1% gelatin-coated plates. Peripheral blood-derived EPCs (C26b, EPC1, and EPC2) were cultured as described (Ormiston et al., 2015) in endothelial cell basal medium (PromoCell, c-22210) supplemented with 10% FBS and cytokines, without heparin.

### RNA Reprogramming

Reprogramming was performed using the StemRNA 3rd Gen Reprogramming Kit (Stemgent, 00-0076). A detailed protocol is provided in Supplemental Information. In brief, fibroblasts were plated on Geltrex in culture medium with serum. The following day, RNAs were delivered by lipofectamine RNAiMAX (Thermo Fisher Scientific, 13778150) and transfection repeated daily for 3–4 days in medium supplemented with FGF2. From day 7, cultures were exchanged to naive culture medium until naive-type colonies formed. For EPC reprogramming, mRNA cocktails were delivered daily for 8 days in EPC expansion medium. The culture was then switched to PXGL plus Y-27632 medium for 14–20 days until dome-shaped colonies became pronounced.

### hPSC Differentiation

Naive hPSC capacitation and tri-lineage differentiation were performed as described previously (Rostovskaya et al., 2019). In brief, naive hPSCs were capacitated for more than 10 days to prepare them for lineage induction. Definitive endoderm was induced over 3 days: day 1 in CDM2 basal medium supplemented with 100 ng/mL, activin A, 100 nM PI-103, 3 μM CHIR99021, 10 ng/mL FGF2, 3 ng/mL BMP4, 10 μg/mL heparin, and followed by 2 days in CDM2 supplemented with 100 ng/mL activin A, 100 nM PI-103, 20 ng/mL FGF2, 250 nM LDN193189, 10 μg/mL heparin (Loh et al., 2014). Neuroectoderm was induced in N2B27 medium supplemented with 1 μM A83-01 and 500 nM LDN193189 for 10 days (Chambers et al., 2009). Differentiation to paraxial mesoderm was induced for 6 days in 3 μM CHIR99021 and 500 nM LDN193189, with addition of 20 ng/mL FGF2 from days 3 to 6 (Chal et al., 2015).

### Real-Time and RT-PCR

Total RNA was extracted using ReliaPrep Kit (Promega, Z6012) and cDNA synthesized with GoScript reverse transcriptase (Promega, A5004) and oligo(dT) adapter primers. TaqMan assays (Thermo Fisher Scientific) and Universal Probe Library probes (Roche Molecular Systems) were used to perform gene quantification.

### Immunostaining

Cells were fixed with 4% paraformaldehyde for 10 min at room temperature and blocked/permeabilized in PBS with 0.1% Triton X-100, 3% donkey serum for 30 min. Incubation with primary antibodies was overnight at 4°C. Wash was in 0.1% Triton X-100 twice, 10 min each time. Secondary antibodies were added for 1 h at room temperature. The following antibodies were used for immunostaining of pluripotency markers: NANOG (Bio-Techne, AF1997), OCT4 (Santa Cruz, sc-5279), KLF4 (Santa Cruz, sc-20691), KLF17 (Atlas Antibodies, HPA024629), TFCP2L1 (Bio-Techne, AF5726). Antibodies for immunostaining of differentiation markers were: FOXA2 (R&D Systems, AF2400), SOX17 (Bio-Techne, AF1924), SOX1 (Bio-Techne, AF3369), PAX6 (Merck Millipore, AB2237), and TBX6 (Abcam, ab38883). For live staining, cells were incubated with conjugated SUSD2 clone W5C5 (SUSD2-PE, BioLegend, 327406) in culture medium for 20 min before washing and imaging.

### Flow Cytometry

Flow cytometry analysis was carried out on a CyAn ADP (Beckman Coulter) or BD LSRFortessa instrument (BD Biosciences) with analysis using FlowJo software. For intracellular marker staining, cells were fixed with fixation buffer (Thermo Fisher Scientific, 00-8222-49) for 30 min at 4°C, washed with permeabilization buffer (Thermo Fisher Scientific, 00-8333-56), and incubated with SOX17 antibody diluted with permeabilization buffer and 5% donkey serum (Merck, D9663) for 1 h at 4°C. Cell sorting was performed using a MoFlo high-speed instrument (Beckman Coulter). The following antibodies were used for flow cytometry: SUSD2-PE (BioLegend, 327406), CD24-APC (Thermo Fisher Scientific, 17-0247-42), EpCAM-PE/Cy7 (BioLegend, 324221), TRA-1-85-FITC (Miltenyi Biotec, 130-107-106), CXCR4-PE (BD Pharmingen, 555974), and SOX17-APC (Bio-Techne, IC1924A).

### Chromosome Analysis

G-banded karyotype analysis was performed following standard cytogenetics protocols at Sheffield Diagnostic Genetics Service. Typically 20 metaphases were scored. CGH array analysis using the Agilent ISCA 8× 60K v2 array was carried out at the Cytogenetics Laboratory, Cambridge University Hospitals.

### Transcriptome Sequencing and Data Analysis

Naive hPSCs were cultured on Geltrex or 10 μg/cm^2^ laminin (Merck, CC095) without MEFs for three passages before harvesting for RNA. Total RNA was extracted from three biological replicates of each cell line using TRIzol/chloroform (Thermo Fisher Scientific, 15596018) and RNA integrity assessed by Qubit measurement and an RNA nanochip bioanalyzer. Ribosomal RNA was depleted from 1 μg of total RNA using Ribo-Zero (Illumina kit). Sequencing libraries were prepared using the TruSeq RNA Sample Prep Kit (Illumina, RS-122-2001). Sequencing was performed on the Illumina NextSeq 500 High Output Kit v2 (75 cycles) (Illumina, FC-404-1005), according to the manufacturer's instructions.

Reads were aligned to human genome build GRCh38/hg38 with STAR (Dobin et al., 2013) using the human gene annotation from Ensembl release 87 (Yates et al., 2016). Alignments to gene loci were quantified with HTseq-count (Anders et al., 2014) based on annotation from Ensembl 87 and using option –m intersection-nonempty. Fractional identity between *in-vitro*-cultured cells and pre-implantation stages was computed using R package DeconRNASeq (Gong and Szustakowski, 2013) and method as described previously (Stirparo et al., 2018). External datasets used for comparative analyses are detailed elsewhere (Guo et al., 2017, Stirparo et al., 2018). Principal-component analyses were performed based on log_2_ fragments per kilobase of transcript per million mapped reads (FPKM) computed with the Bioconductor packages *DESeq2* (Love et al., 2014) or *FactoMineR* (Lê et al., 2008) in addition to custom scripts.

### Transposable Element Analysis

Reads were trimmed and low-quality bases were removed using *TrimGalore!* (github.com/FelixKrueger/TrimGalore). Quality-trimmed reads were aligned to the human reference genome (UCSC hg38/NCBI GRCh38) using *bowtie* (bowtie-bio.sorceforge.net) with options “-a –best -M 1 -v 2,” allowing for two mismatches and randomly reporting one alignment for multi-mapping reads. RepeatMasker-annotated regions were obtained from the hg38 UCSC Table Browser, and counts per TE were extracted using *featureCounts* (bioinf.wehi.edu.au/featureCounts) requiring at least 10 nt overlap and counting multi-mapping reads. RepeatMasker-annotated TEs with at least five counts over all samples were considered for further analysis. Read counts per TE were normalized and statistical significance for differential expression between all samples was evaluated using the R Bioconductor *DESeq* package (www.bioconductor.org). Expression values were further normalized by the size of TE (per 1 kB). Unsupervised hierarchical clustering was performed using the R *hclust* function.

### Whole-Genome Bisulfite Sequencing, Mapping, and Analysis

Post-bisulfite adaptor-tagging libraries for whole-genome DNA methylation analysis were prepared from purified genomic DNA (Miura et al., 2012, Smallwood et al., 2014, von Meyenn et al., 2016). Paired-end sequencing was carried out on HiSeq 2500 instruments (Illumina). Raw sequence reads were trimmed to remove poor-quality reads and adapter contamination using Trim Galore (v0.4.1) (Babraham Bioinformatics). The remaining sequences were mapped using Bismark (v0.14.4) (Krueger and Andrews, 2011) to the human reference genome GRCh37 in paired-end mode as described previously (von Meyenn et al., 2016). CpG methylation calls were analyzed using SeqMonk software (Babraham Bioinformatics). Global CpG methylation levels of pooled replicates were illustrated using boxplots. The SeqMonk build-in tSNE analysis was used to generate tSNE plots of the various datasets. The genome was divided into consecutive 20-kb tiles, and percentage methylation was calculated using the bisulfite feature methylation pipeline in SeqMonk. Scatterplots of methylation levels over 20-kb tiles were generated using R, highlighting hypermethylated DMRs. Annotations of human germline imprint control regions were obtained as described previously (Court et al., 2014). Pseudocolor heatmaps representing average methylation levels were generated using the R heatmap.2 function without further clustering, scaling or normalization.

## Author Contributions

Conceptualization, G.G. and A.S.; Methodology, G.G.; Investigation, G.G., N.B., J.Y., J.C., D.B., R.D., M.R., C.W., D.L., and Y.L.; Formal Analysis, G.G.S., F.v.M., and S.D.; Writing, A.S. and G.G.; Supervision, S.E.-M., G.G., and A.S.

## Accession Numbers

RNA-seq and WGBS data are deposited in GEO under accession numbers GEO: GSE138304 and GSE130162.

## Supplemental Information

Document S1. Supplemental Experimental Procedures, Figures S1–S5, and Tables S1 and S2

Document S2. Article plus Supplemental Information
