# Supplementary material for: Wnt Inhibition Facilitates RNA-Mediated Reprogramming of Human Somatic Cells to Naive Pluripotency
Source: Stem Cell Reports. 2019 Nov 7;13(6):1083–98. doi: 10.1016/j.stemcr.2019.10.009 (PMC6915845; doi:10.1016/j.stemcr.2019.10.009)
Supplement: Document S1. Supplemental Experimental Procedures, Figures S1–S5, and Tables S1 and S2 [file mmc1.pdf]

Stem Cell Reports, Volume *13*

Supplemental Information

Wnt Inhibition Facilitates RNA-Mediated Reprogramming of Human Somatic Cells to Naive Pluripotency

Nicholas Bredenkamp, Jian Yang, James Clarke, Giuliano Giuseppe Stirparo, Ferdinand von Meyenn, Sabine Dietmann, Duncan Baker, Rosalind Drummond, Yongming Ren, Dongwei Li, Chuman Wu, Maria Rostovskaya, Sarah Eminli-Meissner, Austin Smith, and Ge Guo

Inventory of supplemental information

Supplemental figures and legends

Figure S1. Wnt inhibition enhances naïve reprogramming by RNA, relates to Figure 1
Figure S2. Reproducibility of reprogramming, relates to Figure 2
Figure S3. EPC reprogramming, relates to Figure 3
Figure S4. RT-qPCR analysis of lineage induction, relates to Figure 4
Figure S5. Analysis of transposon element expression and CpG methylation, relates to Figure 5

Supplemental Tables

Table S1. Taqman assays
Table S2. PCR primers and related UPL probes

Supplemental protocol

mRNA reprogramming of HDFs and EPCs.

Figure S1

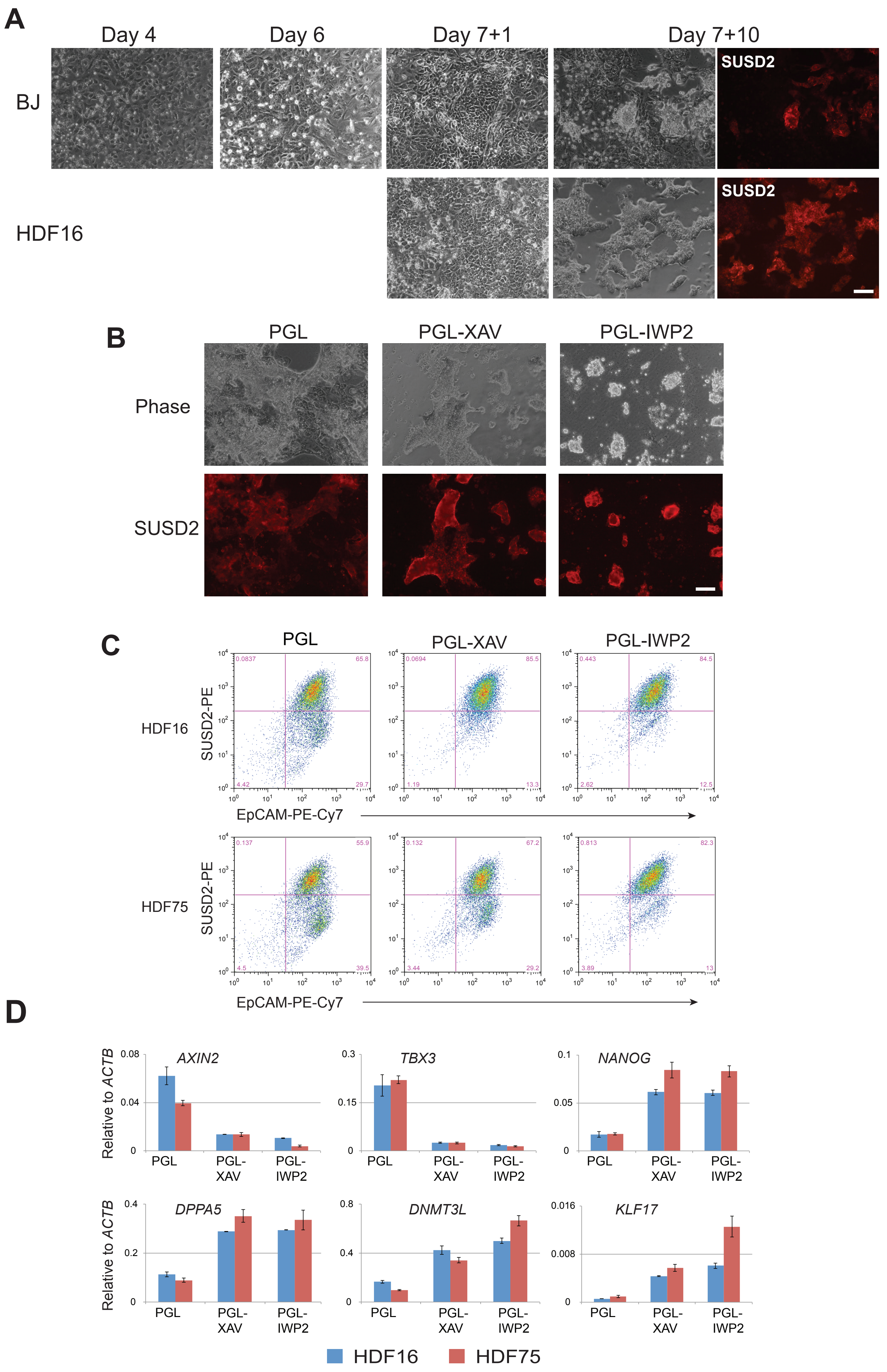
A
Day 4
Day 6
Day 7+1
Day 7+10
BJ
HDF16
SUSD2
SUSD2
B
PGL
PGL-XAV
PGL-IWP2
Phase
SUSD2
C
PGL
PGL-XAV
PGL-IWP2
HDF16
HDF75
SUSD2-PE
EpCAM-PE-Cy7
D
AXIN2
TBX3
NANOG
DPPA5
DNMT3L
KLF17
Relative to ACTB
PGL
PGL-XAV
PGL-IWP2
HDF16
HDF75

Figure S2

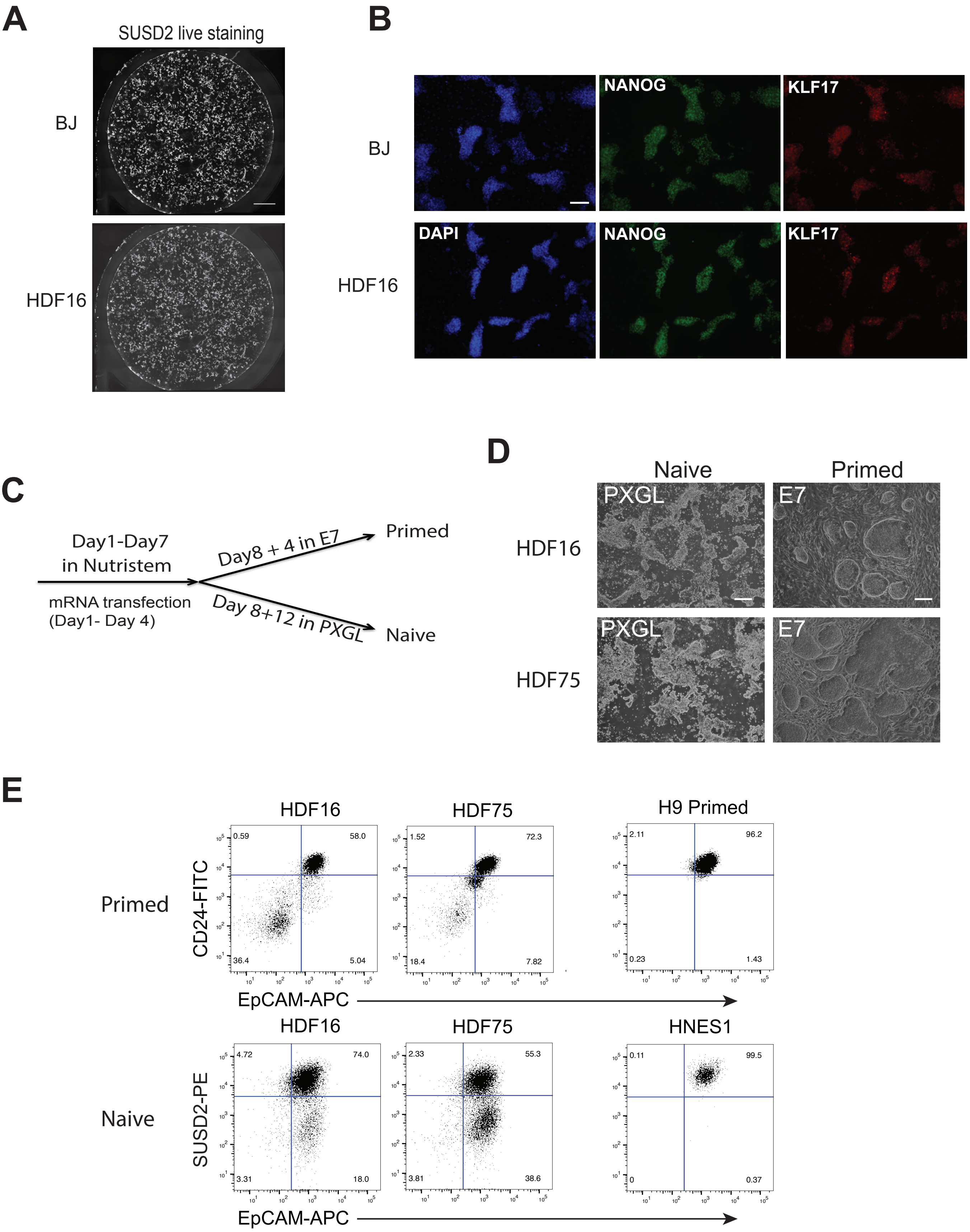
A
SUSD2 live staining
BJ
HDF16
B
BJ
HDF16
DAPI
NANOG
KLF17
C
Day1-Day7
in Nutristem
mRNA transfection
(Day1- Day 4)
Day8 + 4 in E7
Day 8+12 in PXGL
Primed
Naive
D
Naive
Primed
HDF16
HDF75
PXGL
E7
E
Primed
Naive
HDF16
HDF75
H9 Primed
HNES1
CD24-FITC
SUSD2-PE
EpCAM-APC
0.59
58.0
36.4
5.04
1.52
72.3
18.4
7.82
2.11
96.2
0.23
1.43
4.72
74.0
3.31
18.0
2.33
55.3
3.81
38.6
0.11
99.5
0
0.37

Figure S3

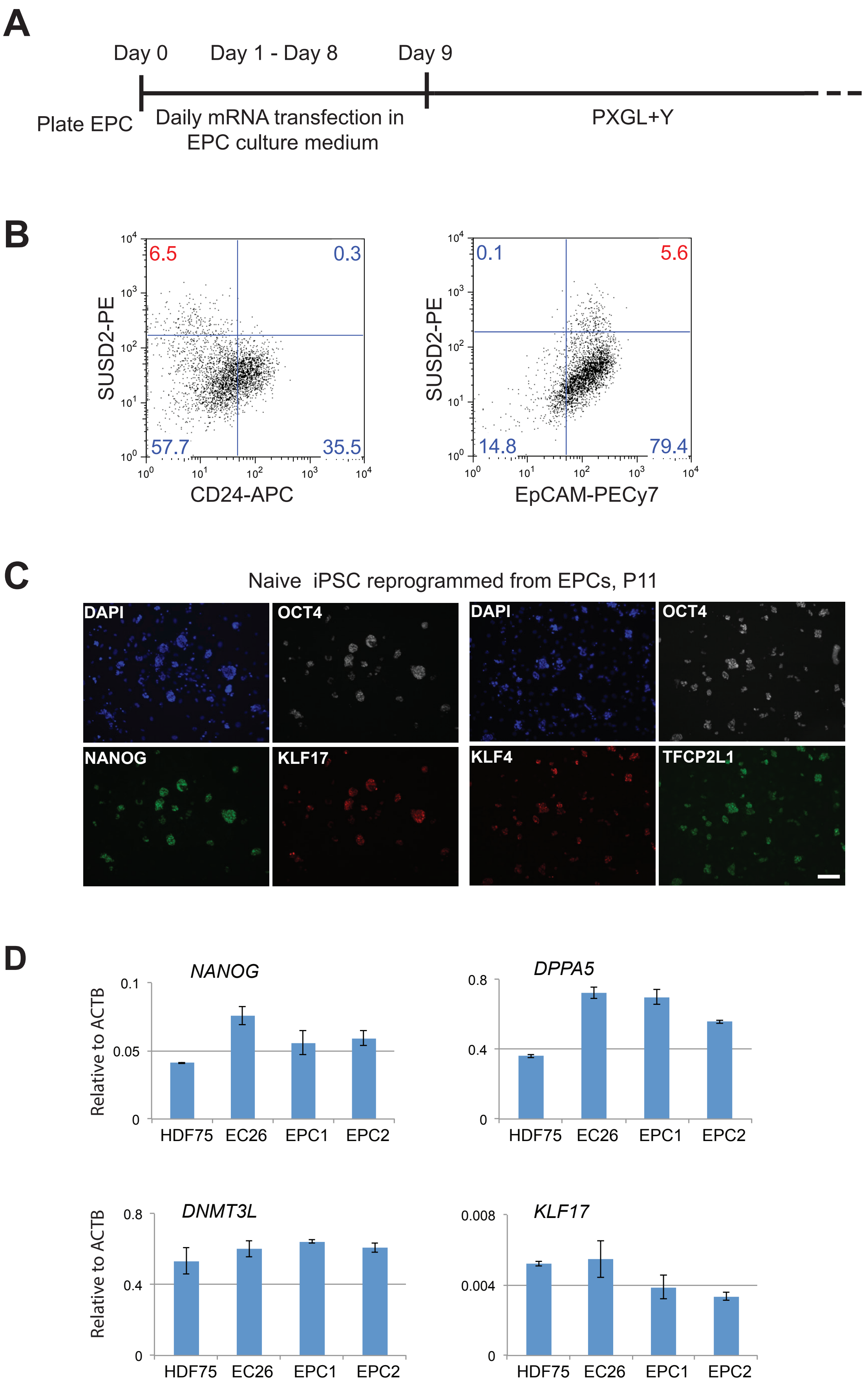
A
Day 0
Day 1 - Day 8
Day 9
Plate EPC
Daily mRNA transfection in EPC culture medium
PXGL+Y
B
SUSD2-PE
CD24-APC
6.5
0.3
57.7
35.5
EpCAM-PECy7
0.1
5.6
14.8
79.4
C
Naive iPSC reprogrammed from EPCs, P11
DAPI
OCT4
NANOG
KLF17
KLF4
TFCP2L1
D
NANOG
DPPA5
DNMT3L
KLF17
Relative to ACTB
HDF75
EC26
EPC1
EPC2

Figure S4

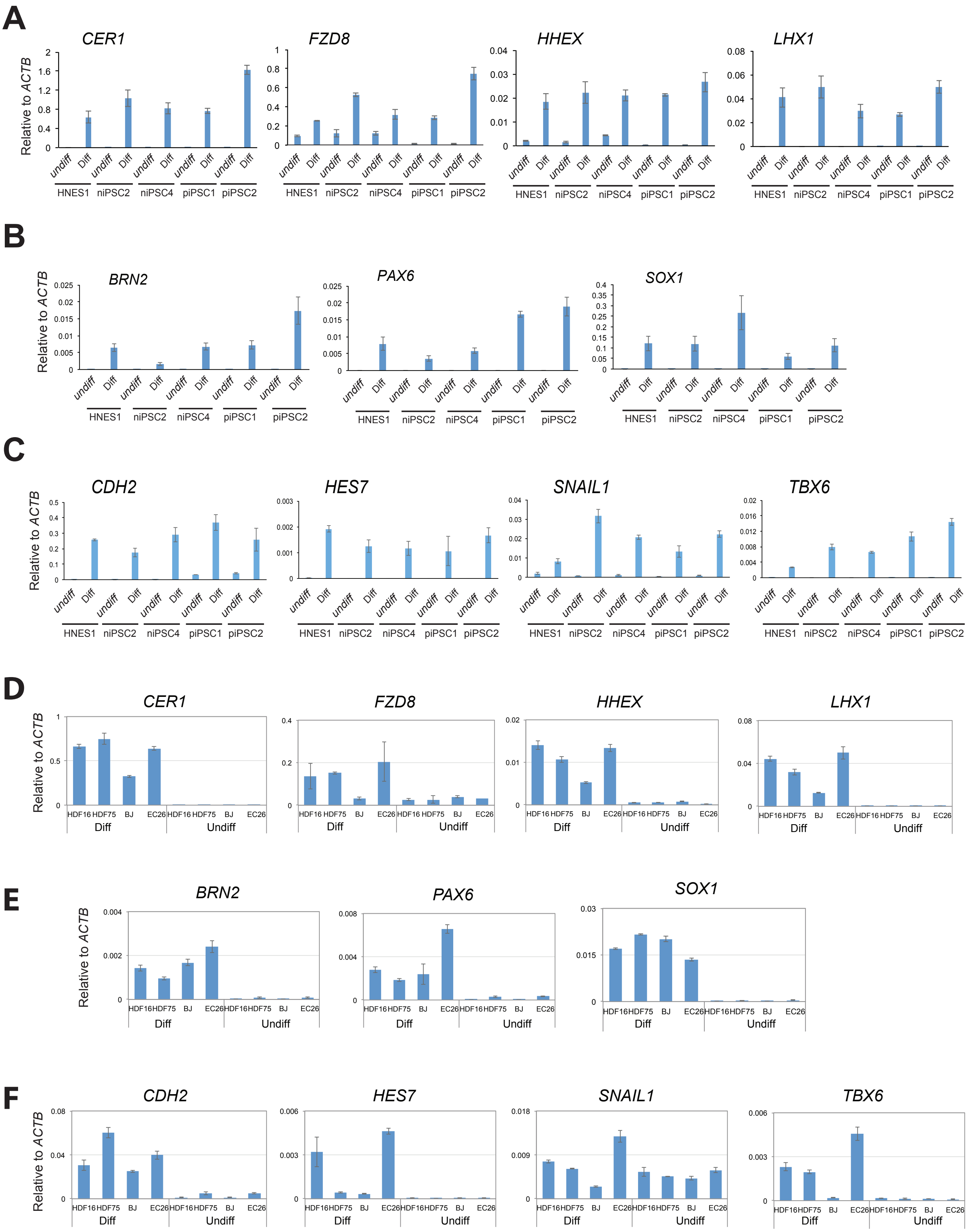
A
CER1
FZD8
HHEX
LHX1
Relative to ACTB
undiff
Diff
HNES1
niPSC2
niPSC4
piPSC1
piPSC2
B
BRN2
PAX6
SOX1
C
CDH2
HES7
SNAIL1
TBX6
D
E
F
HDF16
HDF75
BJ
EC26
Undiff

Figure S5

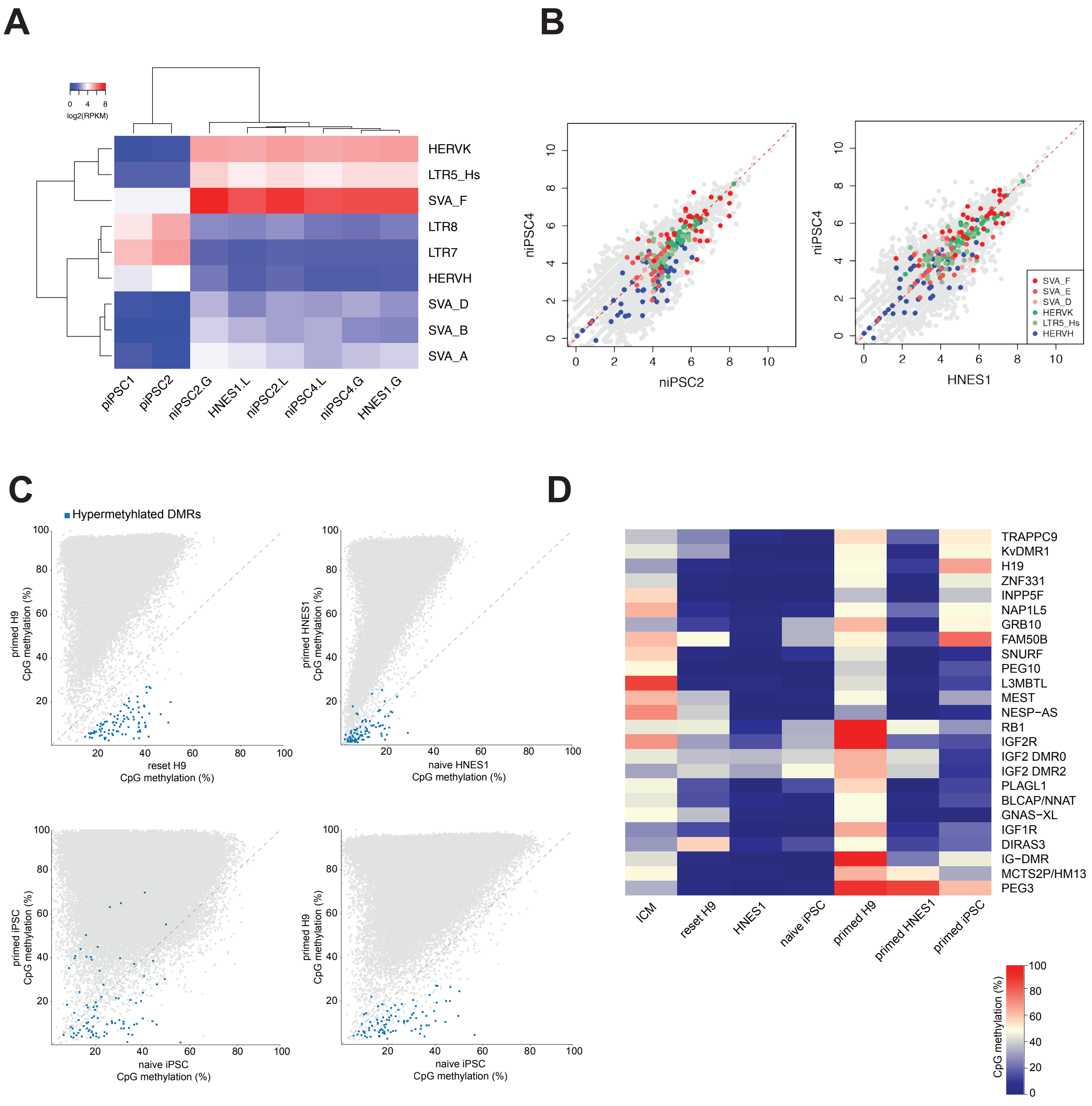
A
B
C
D
log2(RPKM)
HERVK
LTR5_Hs
SVA_F
LTR8
LTR7
HERVH
SVA_D
SVA_B
SVA_A
piPSC1
piPSC2
niPSC2.G
HNES1.L
niPSC2.L
niPSC4.L
niPSC4.G
HNES1.G
niPSC4
niPSC2
HNES1
SVA_F
SVA_E
SVA_D
HERVK
LTR5_Hs
HERVH
Hypermetyhlated DMRs
primed H9 CpG methylation (%)
reset H9 CpG methylation (%)
primed HNES1 CpG methylation (%)
naive HNES1 CpG methylation (%)
primed iPSC CpG methylation (%)
naive iPSC CpG methylation (%)
TRAPPC9
KvDMR1
H19
ZNF331
INPP5F
NAP1L5
GRB10
FAM50B
SNURF
PEG10
L3MBTL
MEST
NESP−AS
RB1
IGF2R
IGF2 DMR0
IGF2 DMR2
PLAGL1
BLCAP/NNAT
GNAS−XL
IGF1R
DIRAS3
IG−DMR
MCTS2P/HM13
PEG3
ICM
reset H9
HNES1
naive iPSC
primed H9
primed HNES1
primed iPSC
CpG methylation (%)

Supplemental Figure Legends

Figure S1. Wnt inhibition enhances naïve reprogramming by RNA, relates to Figure 1

S1A. Morphology BJ and HDF16 during reprogramming
S1B. Images of HDF75 reprogramming culture in naïve capture medium, PGL and PGL with Wnt inhibitor, XAV939 (XAV) or IWP2.
S1C. Flow cytometry analysis of EpCAM and SUSD2 expression after 12 days in PGL with XAV or IWP2.
S1D. RT-qPCR analysis of markers after 12 days in PGL based medium.
Scale bar, 100 μM. Error bars indicate s.d. of two technical replicates.

Figure S2. Reproducibility of reprogramming, relates to Figure 2

S2A. Wells of reprogramming cultures after 13 days in PXGL, stained in situ with SUSD2-PE antibody. Scale bar, 2 mm
S2B. Immunostaining for KLF17 and NANOG after 15 days in PXGL. Scale bar, 100 μM
S2C. Schematic of reprogramming to primed and naïve iPSCs by RNA.
S2D. Morphology of HDF16 and HDF75 after reprogramming to naïve and primed iPSCs. Scale bar, 100 μM
S2E. Flow cytometry analysis of reprogramming to primed and naïve iPSCs. H9 primed and HNES1 are included as control for primed and naïve ESCs.

Figure S3. EPC reprogramming, relates to Figure 3
S3A. Schematic of EPC reprogramming protocol
S3B. Flow cytometry analysis of SUSD2, CD24 and EpCAM expression after three weeks in PXGL.
S3C. Immunostaining of pluripotency markers in expanded EPC-derived naive iPSCs. Scale bar, 100 μM
S3D. RT-qPCR analysis of three EPC derived niPSC cultures (EC26, EPC1, EPC2), comparing to HDF75 derived niPSCs. Error bars indicate s.d. of two technical replicates.

Figure S4. RT-qPCR analysis of lineage induction, relates to Figure 5
S4A. Definitive endoderm induction of naive iPSCs (niPSC2, niPSC4) and primed iPSCs (piPSC1, piPSC2)
S4B. Neuroectoderm induction of naive iPSCs (niPSC2, niPSC4) and primed iPSCs
S4C. Paraxial mesoderm induction of naive iPSCs (niPSC2, niPSC4) and primed iPSCs
S4D. Definitive endoderm induction of naïve iPSCs derived from HDFs and EPC
S4E. Neuroectoderm induction of of naïve iPSCs derived from HDFs and EPC
S4F. Paraxial mesoderm induction of naive iPSCs derived from HDFs and EPC
Error bars indicate s.d. of three technical replicates.

Figure S5. Analysis of transposon element expression and CpG methylation, relates to Figure 6
S5A. A heatmap showing the expression of known naive and primed-specific TEs (average expression of all TE loci of the subfamily).
S5B. Scatter plots showing the expression of TE elements in niPSC2, C4 and HNES1 cells. TE elements from representative TE subfamilies that are differentially expressed between naïve and primed cells (Theunissen et al., 2016, Guo et al., 2017) are highlighted.
S5C. Scatter plots of CpG methylation percentages over tiles spanning 20 kb. Regions with >10% gain in CpG methylation in reset H9-NK2 cells[9] compared to conventional primed H9 cells are highlighted in blue in all scatterplots.
S5D. Averaged CpG methylation of known DMRs of imprinted maternal and paternal genes.

Table S1. Taqman assays, relates to experimental procedures

Gene	TaqMan Assay ID
ACTB	Hs01060665_g1
NANOG	Hs02387400_g1
OCT4	Hs01654807_s1
KLF4	Hs00358836_m1
KLF17	Hs00703004_s1
TFCP2L1	Hs00232708_m1
DPPA3	Hs01931905_g1
DPPA5	Hs00988349_g1
DNMT1	Hs00945875_m1
DNMT3A	Hs01027166_m1
DNMT3B	Hs00171876_m1
DNMT3L	Hs01081364_m1
SOX2	Hs01053049_s1
PRDM14	Hs01119056_m1

Table S2. PCR primers and related UPL probes, relates to experimental procedures

Gene	Forward Primer	Reverse Primer	UPL probe
SOX1	accaggccatggatgaag	cttaattgctggggaattgg	37
PAX6	ggcacacacacattaacacactt	ggtgtgtgagagcaattctcag	9
BRN2	aataaggcaaaaggaaagcaact	caaaacacatcattacacctgct	57
HHEX	cggacggtgaacgactaca	agaaggggctccagagtagag	61
LHX1	atgcaacctgaccgagaagt	caggtcgctaggggagatg	80
CER1	gccatgaagtacattgggaga	cacagccttcgtgggttatag	41
FZD8	cgccacgcgttaatttct	ccggttctggaaccacac	19
CDH2	tgcacagatgtggacaggat	ccacaaacatcagcacaagg	15
SNAI1	gcgagctgcaggactctaat	cggtggggttgaggatct	62
HES7	gcagcctggaagagctga	acggcgaactccaatatctc	78
TBX6	gaacggcagaaactgtaagagg	gtgtgtctccgctcccatag	5
TCF15	tgttccgggacactctgg	caggctgaatggatcctcac	80
ZEB1	agcacttaagaattcacagtggag	catttcttactgcttatgtgtgagc	36
OTX2	gggtatggacttgctgcac	ccgagtgaacgtcgtcct	81
TBX3	ggtcattaccaagtcgggaag	tcagcagctataatgtccatcaa	26

Supplemental protocol

1. Reprogramming human dermal fibroblasts to naïve pluripotent stem cells

Materials

HDFa (human dermal fibroblast, adult)
Irradiated mouse embryonic fibroblast (MEF)
StemRNA 3rd Gen Reprogramming Kit (Stemgent,00-0076)
Lipofectamine® RNAiMAX™ (Thermo Fisher Scientific, 13778150)
Geltrex (hESC-Qualified, Thermo Fisher Scientific, A1413302)
SUSD2 (PE conjugate) (BioLegend, 327406)

Culture media

Fibroblast culture medium
DMEM high glucose (Merck, D5546), FBS (10%, Merck, F0804), L-glutamine (2 mM, Thermo Fisher Scientific, 25030-024), 2-mercaptoethanol (100 µM, Merck, M3148)

Modified E7 medium
Home made E6 basal medium (Chen et al., 2011) supplemented with 10 ng/mL FGF2 (prepared in-house).

NutriStem^{TM} hPSC XF Medium (Biological Industries, 01-0005)

Naïve hPSC medium, PXGL
N2B27 medium supplemented with MEK inhibitor PD0325901 (1 µM), tankyrase inhibitor XAV939 (2 µM), aPKC inhibitor Gö6983 (2 µM), human LIF (10 ng/mL, prepared in-house), and optionally Rho-kinase inhibitor Y-27632 (10 µM).

Note. The quality of N2B27 medium is of paramount importance and batches should be tested on naive hPSCs if available, or else on mouse ES cells as described (Mulas et al., 2019)

Protocol

1: Day 0: Dissociate HDFs with TrypLE. Collect dissociated cells, pellet at 300g for 3 minutes and resuspend in fibroblast culture medium. Count cells and plate at a density of $1x10^4/cm^2$ on tissue culture plates pre-coated with Geltrex (1 µL/cm^2).
2: Day 1: Switch to modified E7 medium and perform mRNA transfection following recommendation of StemRNA™-NM Reprogramming Kit protocol.
3: Day 2-4: Repeat mRNA transfection daily. **Note**: If excessive cell death is observed after mRNA transfection, it is recommended to plate fibroblasts at higher density. Alternatively, reducing mRNA transfection to 3 days is usually sufficient to generate at least 20 naïve colonies/4-well dish.
4: Day 5-6: Refresh culture with modified E7 medium. NutriStem^{TM} can be used as an alternative medium. **Note,** by day 6, patches of cells with epithelial morphology should become apparent, indicating reprogramming has been initiated.
5: Day 7: Switch to human naïve culture medium, PXGL, and maintain for about two weeks. SUSD2 positive colonies should appear after 7-10 days in PXGL medium and can be visualised by live cell staining (Bredenkamp et al., 2019). Rock inhibitor (Y-27632) may be added to PXGL medium during reprogramming. **Note**, transfer to PXGL medium can be varied between Day 6 to Day 9. Delaying medium switch beyond Day 10 will significantly reduce reprogramming efficiency.

2. Reprogramming human blood outgrowth derived endothelial progenitor cells (EPC) to naïve pluripotent stem cells

Materials

EPC reprogramming uses the same materials as HDFa reprogramming, if not specified otherwise.

EPC medium (50 mL)
Endothelial Cell basal medium (PromoCell, c-22210) or EBM-2
Endothelial Cell basal medium (Lonza, cc-3156) 40-45 mL
5-10 mL heat-inactivated FBS (heat-inactivation is not necessary) (20% FBS when thawing the EPCs, 10% FBS for regular culture)
Hydrocortisone (Lonza, cc-4112A) 16 µL
hFGF-B (Lonza, cc-4113A) 160 µL
VEGF (Lonza, cc-4114A) 16 µL
R3-IGF-1 (Lonza, cc-4115A) 16 µL
hEGF (Lonza, cc-4317A) 16 µL
Ascorbic acid (Lonza, cc-4116A) 16 µL
GA-1000 (Lonza, cc-4381A) 16 µL

Reprogramming EPCs
1. EPCs are cultured according to (Ormiston et al., 2015). Cells are grown in 50 µg/mL Collagen I coated T-75 flasks with endothelial growth medium supplemented with growth factors with 10-20% FBS and without heparin (EPC medium).
2. When EPCs reach 80-90% confluence, cells are dissociated with TrypLE then resuspended at $2x10^6$/mL in EPC medium.
3. Add 1-$2x10^5$ EPCs (0.5 mL) per well of a 4-well dish (coated with 2.4 µg/mL Laminin 511 (iMatrix-511, Reprocell, T303) at least 1 h before plating).
4. Next day, refresh with EPC medium about one hour before transfection. We normally do mRNA transfection after 5pm.
5. Perform mRNA cocktail transfection as detailed in *StemRNA 3rd Gen Reprogramming Kit.*
6. Next morning usually before 9-9.30am, refresh culture with fresh EPC medium; then late in the afternoon repeat mRNA transfection.
7. Repeat daily until Day 9.
8. Day 9, exchange to human naïve medium with 10 µM Y-27632 for naïve iPSC induction.
9. Following 15-20 days culture in naïve medium, naïve colonies should be readily identifiable by refractile dome-shaped morphology. At this point, naïve colonies can be picked or bulk passaged.

3. Passaging naïve cells

1. Dissociate culture with Accutase or TrypLE Express (about 5-10 minutes at 37^0C).
2. Pellet cells at 300g for 3 min. Aspirate and re-suspend cells in PXGL with Y-27632 (PXGLY).
3. Aliquot cells to plates coated with MEF feeders in PXGL plus Y-27632. We recommend adding Geltrex (0.5 µL per cm^2) to cells at the time of passaging.
4. The next day, top up wells with fresh PXGL medium (without Y-27632). Subsequently, change half-medium daily until passaging. This normally takes 4-5 days culture in PXGL medium at a split ratio of 1:4 to 1:8. Do not let colonies grow too large.

Note: A stable naïve iPSC culture will present with more than 80% SUSD2+CD24- cells after three passages in PXGL medium. If not, picking colonies or sorting for SUSD2+CD24- may be necessary to establish a stable iPSC culture.

References:
Bredenkamp, N., Stirparo, G.G., Nichols, J., Smith, A., and Guo, G. (2019). The Cell-Surface Marker Sushi Containing Domain 2 Facilitates Establishment of Human Naive Pluripotent Stem Cells. Stem Cell Reports *12*, 1212-1222.
Chen, G., Gulbranson, D.R., Hou, Z., Bolin, J.M., Ruotti, V., Probasco, M.D., Smuga-Otto, K., Howden, S.E., Diol, N.R., Propson, N.E., *et al.* (2011). Chemically defined conditions for human iPSC derivation and culture. Nat Methods *8*, 424-429.

Mulas, C., Kalkan, T., von Meyenn, F., Leitch, H.G., Nichols, J., and Smith, A. (2019). Defined conditions for propagation and manipulation of mouse embryonic stem cells. Development *146*, dev173146

Ormiston, M.L., Toshner, M.R., Kiskin, F.N., Huang, C.J., Groves, E., Morrell, N.W., and Rana, A.A. (2015). Generation and Culture of Blood Outgrowth Endothelial Cells from Human Peripheral Blood. J Vis Exp, e53384.
